# RNA-binding residues prediction using structural features

**DOI:** 10.1186/s12859-015-0691-0

**Published:** 2015-08-09

**Authors:** Huizhu Ren, Ying Shen

**Affiliations:** 1 2011 Collaborative Innovation Center of Tianjin for Medical Epigenetics, Key Laboratory of Hormones and Development (Ministry of Health), Metabolic Diseases Hospital & Tianjin Institute of Endocrinology, Tianjin Medical University, Tianjin, 300070 China; 2 School of Software Engineering, Tongji University, Shanghai, 201804 China; 3 Key Laboratory of Intelligent Perception and Systems for High-Dimensional Information, Ministry of Education, Nanjing University of Science and Technology, Nanjing, 210094 P.R. China

**Keywords:** Protein-RNA interaction prediction, Structural information, Least-squares distance

## Abstract

**Background:**

RNA-protein complexes play an essential role in many biological processes. To explore potential functions of RNA-protein complexes, it’s important to identify RNA-binding residues in proteins.

**Results:**

In this work, we propose a set of new structural features for RNA-binding residue prediction. A set of template patches are first extracted from RNA-binding interfaces. To construct structural features for a residue, we compare its surrounding patches with each template patch and use the accumulated distances as its structural features. These new features provide sufficient structural information of surrounding surface of a residue and they can be used to measure the structural similarity between the surface surrounding two residues. The new structural features, together with other sequence features, are used to predict RNA-binding residues using ensemble learning technique.

**Conclusions:**

The experimental results reveal the effectiveness of the proposed structural features. In addition, the clustering results on template patches exhibit distinct structural patterns of RNA-binding sites, although the sequences of template patches in the same cluster are not conserved. We speculate that RNAs may have structure preferences when binding with proteins.

## Background

RNA-protein complexes play an essential role in many biological processes, such as RNA splicing [1], translation [2, 3] and post-transcriptional gene regulation [4, 5], etc. Many large ribonucleoproteins (e.g. ribosome) are also RNA-protein complexes. In addition, certain proteins carry out specific functions such as repairing damaged RNAs [6] and editing transcribed RNAs [7]. Currently, there are 1,542 RNA-binding proteins in the human body which have been manually curated [8].

To understand the molecular mechanisms of the protein-RNA recognition, it is important to identify RNA-binding residues from target proteins. Obviously, the identification is straightforward if structures of RNA-protein complexes have been known in advance. However, it is expensive and time consuming to determine the structure of an RNA-protein complex through biological experiments. Considering this, people are resorting to computational methods which can quickly and accurately predict RNA-binding residues.

In previous studies, RNA-binding residues prediction was mostly based on sequence features of proteins. It is because that protein sequences are much easier to obtain than their structures in RNA-protein complexes. For sequence-based methods, the commonly used features include position-specific scoring matrix (PSSM) [9–14], hydrophobicity [15], electrostatics [16, 17], side chain environment [13, 18, 19], residue interface propensity [19–21], and residue accessibility [12, 15].

In the last decades, abundant of 3D structures of RNA-protein complexes are emerging. According to the records in Protein Data Bank [22], there are only 491 RNA-protein complexes deposited before 2010. By August 2014, the number has been dramatically increased to 1560. As a consequence, researchers begin to explore new structural features which can improve the accuracy of RNA-binding residue prediction.

Compared with the efforts spent on exploring sequence features, studies focused on structural features are rather limited [23]. Kim et al. [24] proposed a new structural feature, namely residue doublet interface propensity. It describes the pairing preference of amino acids in protein-RNA interfaces. Chen and Lim [16] predicted RNA-binding residues based on irregular surface patches and clefts on the target proteins. The irregular surfaces and clefts were composed by the most conserved and electrostatically stabilized residues. Therefore, these surface patches and clefts could provide useful RNA-binding information and consequently improve the prediction accuracy. In [25], spatial patches and topological patches on protein surfaces, which were represented as contact graphs, were used to predict RNA-binding residues for target proteins. Instead of proposing new structural features, Towfic et al. [26] adopted several existing structural features, which included surface roughness of a residue [27] and the CX value [28]. Another method, DRNA [29], took a different strategy of using structural information to predict RNA-binding sites. It first aligned the structure of the target protein to the template proteins from an RNA-protein complex library and then predicted RNA-binding residues based on results of structure alignment. There were also some works focusing on utilizing secondary structure elements [30].

In the framework of RNA-binding residue prediction, a protein residue can be represented by a feature vector which is composed by a set of sequence and/or structural features. For protein residues whose binding status have been determined, their feature vectors can be constructed and used to train classification models. RNA binding propensity or status of a target residue can be predicted by the well trained classification models. Currently, a number of classification models have been adopted in RNA-binding residue prediction. One popular classification model is support vector machine (SVM) which has been adopted by several powerful servers such as BindN+, PiRaNhA, etc. [12–14, 18, 31–33]. Other popular classifiers include Naïve Bayes classifier [34, 35] and neural network [30, 36, 37]. In addition to using a single classifier, ensemble classification models which take the advantage of multiple classifiers are also exploited and they have been shown to greatly improve the prediction accuracy [15]. Besides using sequence/structural features, certain methods predict RNA-binding residues directly from amino acid sequences [34, 38].

Protein-RNA interaction interfaces are reported to be composed by several clusters of positive charged residues scattered on protein surfaces [17, 39]. Besides positive charged residues on the protein surface, protein-RNA interfaces also comprise binding pockets/cavities [16, 19]. We assume that RNA-binding sites are assembled by certain patches with specific shapes, which are regarded as binding units. Based on this assumption, we compile a set of template patches which participate in protein-RNA interactions. The templates are extracted from surfaces around RNA-binding residues from the training set. They are further grouped into several clusters. Representative patches, which are centers of clusters, are identified. Based on the representative patches, a set of new structural features can be constructed for a residue. We first extract surface patches around the residue. Then we compare its surface patches with each representative patch and use the accumulated distances as structural features. The dimension of structural features is the same as the number of representative patches. These new features provide sufficient structural information of surrounding surface of a residue. The structural similarity between the environments of two residues can be transformed into the similarity between their structural features. If distances between a target residue and RNA-binding residues are small, it is more likely to bind RNA molecules. If not, it tends to be a non-RNA-binding residue. These new features, together with other features such as PSSM and residue propensity, are used to predict RNA-binding residues. Using machine learning techniques, specifically the ensemble learning technique, the combined features exhibit a good discrimination power for RNA-binding residue prediction.

## Results and discussion

### Experimental results

Proteins in the dataset are divided into four groups and a 4-fold cross validation is adopted to estimate the prediction performance using the new structural features as well as other sequence features (e.g. PSSM scores, residue interface propensity). In each fold, one group is used as the test set and the other three are combined as the training set. An ensemble classifier is trained using feature vectors and class labels (binding or non-binding) of residues from the training set. RNA-binding scores are computed for residues in the test set using their feature vectors by the well-trained classifier.

In addition to evaluating the prediction performance using all features (i.e. the new structural features, PSSM scores, and interface propensity), we evaluate the prediction performance using different combinations of features, i.e. using structural features, PSSM scores, and the combined features which include structural features and PSSM scores. The prediction performances using different combinations of features are shown in Table 1. The performance using PSSM scores is slightly better than using structural features in terms of area under the curve (AUC) and Matthews correlation coefficient (MCC) (AUC_PSSM_ = 0.64 and MCC_PSSM_ = 0.19; AUC_StructuralFeatures_ = 0.62 and MCC_StructuralFeatures_ = 0.18). However, based on some other performance metrics, structural features outperform PSSM scores. For example, the precision for structural features is 0.46 while for PSSM scores it is 0.41.

**Table 1:** Prediction performances using different features

Type of features	AUC	MCC	Precision	Accuracy	Sensitivity	Specificity	F-score
Structural features	0.62	0.18	0.46	0.66	0.38	0.79	0.42
PSSM scores	0.64	0.19	0.41	0.59	0.62	0.58	0.49
Structural features + PSSM scores	0.67	0.24	0.46	0.66	0.58	0.71	0.50
All features	0.68	0.26	0.48	0.68	0.48	0.76	0.48

Comparison of prediction performances using different features

When combining two types of features together, the performance can be greatly improved. Compared with using PSSM scores, the AUC value of using combined features is increased from 0.64 to 0.67. The MCC value is increased from 0.19 to 0.24. When the feature of residue interface propensity is introduced, the prediction performance is slightly improved. The AUC value is increased from 0.67 to 0.68. The MCC value is increased from 0.24 to 0.26. Precision, accuracy, and specificity are also increased from 0.46/0.66/0.71 to 0.48/0.68/0.76, respectively. However, the values of sensitivity and F-score are decreased.

We also evaluated our method on different categories of proteins. The corresponding scores of different performance metrics are shown in Table 2. When evaluated by AUC scores, our method achieves better performances on aptermer, ribosomal, and small classes, on which AUC values are larger than 0.68. When evaluated using MCC values, our method achieves better performances on aptermer and splicing classes, on which MCC values are larger than 0.26.

**Table 2:** Prediction performances on different protein categories

Protein categories	AUC	MCC	Precision	Accuracy	Sensitivity	Specificity	F-score
RNAse	0.65	0.22	0.41	0.66	0.53	0.71	0.46
SRP	0.51	0.11	0.70	0.66	0.04	0.99	0.08
Aptamer	0.71	0.31	0.51	0.78	0.39	0.89	0.44
dsRNA	0.63	0.17	0.31	0.73	0.37	0.81	0.34
Exosome	0.62	0.16	0.27	0.47	0.80	0.37	0.40
mRNA	0.63	0.15	0.26	0.44	0.84	0.32	0.40
Ribosomal	0.68	0.24	0.83	0.63	0.64	0.62	0.72
Small	0.69	0.11	0.22	0.49	0.69	0.44	0.34
snRNP	0.50	0.06	0.38	0.50	0.62	0.44	0.47
Splicing	0.66	0.35	0.60	0.81	0.33	0.94	0.43
tRNA	0.61	0.15	0.31	0.51	0.74	0.44	0.44
Viral	0.59	0.11	0.27	0.41	0.84	0.27	0.41
Other	0.58	0.11	0.23	0.65	0.46	0.68	0.31

Prediction performances on different protein categories

**Fig. 1:**
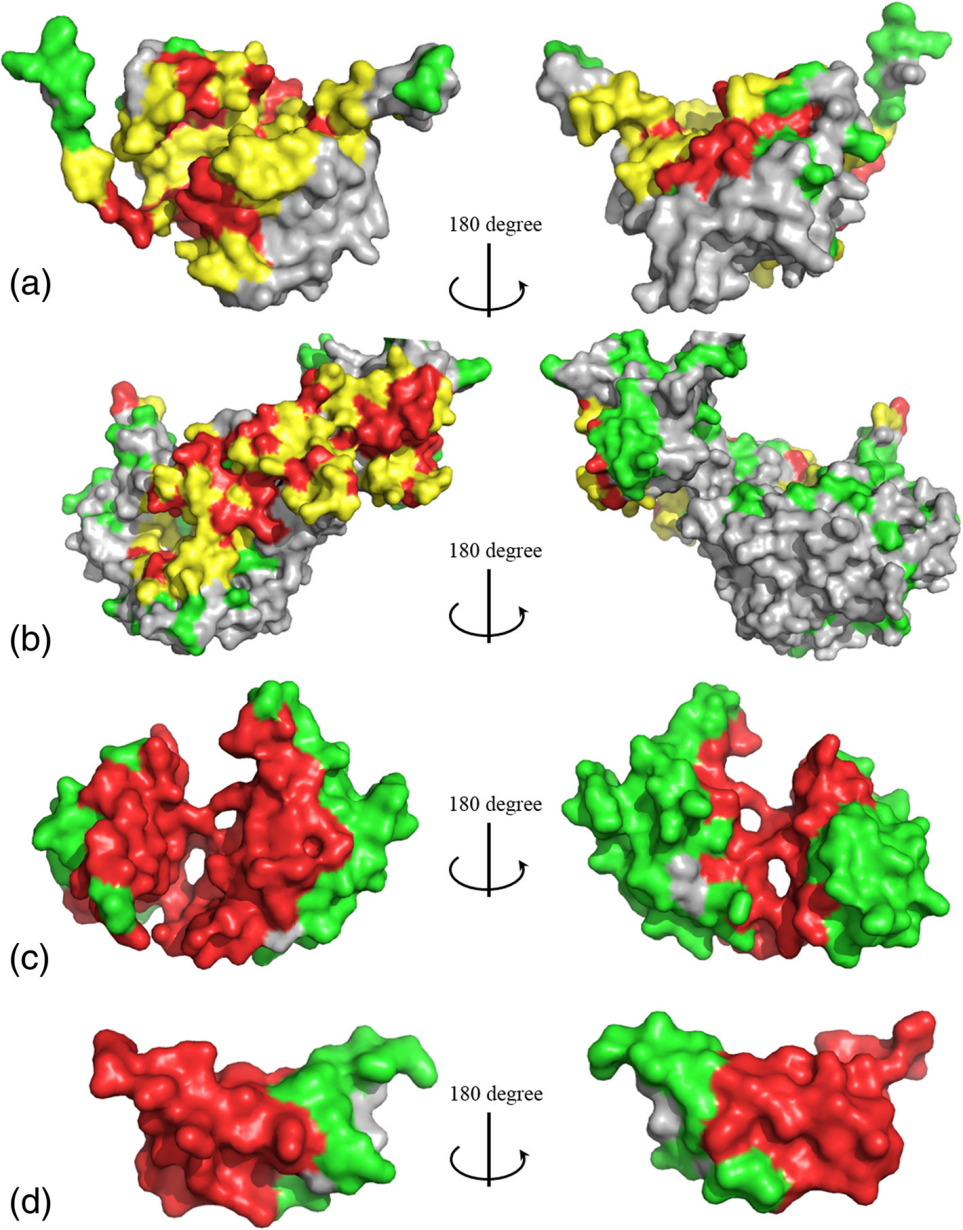
Examples of RNA-binding residue prediction results. RNA-binding residue prediction for (**a**) 4J1G:D (viral), (**b**) 1ASY:A (tRNA), (**c**) 1FXL:A (mRNA), and (**d**) 3 V24:V (ribosomal RNA). True positive, false positive, false negative and true negative residues are shown in red, green, yellow and grey, respectively. (**a**) The numbers of tp, fp, fn, and tn in 4J1G:D are 38, 21, 73, and 95, respectively; (**b**) the numbers of tp, fp, fn, and tn in 1ASY:A are 61, 89, 89, and 251, respectively; (**c**) the numbers of tp, fp, fn, and tn in 1FXL:A are 85, 79, 0, and 3, respectively; (**d**) the numbers of tp, fp, fn, and tn 3 V24:V are 34, 17, 0, and 2, respectively

We select four types of proteins to show the prediction results of our method. Proteins 4J1D:D, 1ASY:A, 1FXL:A, and 3 V24:V belong to viral, tRNA, mRNA, and ribosomal classes, respectively. Figure 1 shows the prediction results of our method for four proteins. Residues of true positive, false positive, false negative and true negative are shown in red, green, yellow, and grey, respectively. The numbers of true positives, false positives, false negatives and true negatives in each protein is also given in the caption of Fig. 1. It can be seen that, most RNA-binding sites in the above four proteins are successfully identified by our method.

### Comparison with other methods

**Fig. 2:**
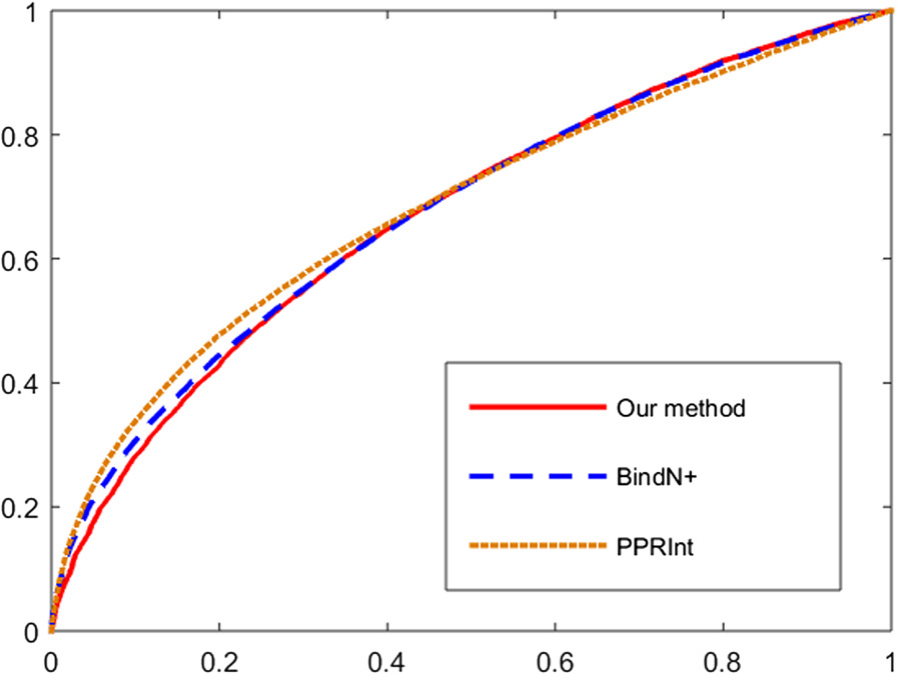
Performance comparison using ROC curves. ROC curves of prediction results of our method, BindN+, and PPRInt

To show the effectiveness of our method, we make a comparison with another three publicly available methods (BindN+ [13], PPRInt [14], and DRNA [29]). These methods showed better performances over other seven methods evaluated by Puton et al. [23]. We evaluate their performances on a new dataset, namely RB344 of PRIDB dataset [40]. RB344 is a non-redundant dataset which is much larger than the dataset used in [23]. The prediction performances of different methods evaluated using proteins from RB344 dataset are listed in Table 3. Receiver operating characteristic (ROC) curves are shown in Fig. 2.

**Table 3:** Performance comparison

Method	AUC	MCC	Precision	Accuracy	Sensitivity	Specificity	F-score
Our method	0.68	0.26	0.48	0.68	0.48	0.76	0.48
DRNA	NA	0.22	0.54	0.75	0.21	0.94	0.31
BindN+	0.68	0.26	0.56	0.72	0.32	0.89	0.41
PPRInt	0.68	0.28	0.53	0.70	0.45	0.82	0.49

Comparison of prediction performances between our method and other three methods

The AUC values of BindN+, PPRInt, and our method are the same, which is 0.68. When evaluated using MCC metric, BindN+ shows the best performance with its value 0.28. Our method and PPRInt have the same MCC value 0.26, which is a bit worse than BindN+. DRNA achieves the worst performance with its value of 0.22. BindN+ has the highest precision and F-score and DRNA has the highest accuracy and specificity. Our method has the highest sensitivity.

## Conclusions

RNA-binding residue prediction is essential for understanding mechanisms of RNA recognition for proteins. In this work, we propose a set of new structural features for RNA-binding residues prediction. Specifically, we construct a set of 3-aa and 2-aa template patches around binding residues and group them into 60 clusters (40 clusters of 3-aa patches and 20 clusters of 2-aa patches). Representative patches which are centers of 60 clusters are identified and used for construct structural features. For each residue, its surrounding patches are extracted. Accumulated distances of surrounding patches to representative patches are computed and comprise a set of structural features. These new structural features, together with other features such as PSSM score and residue interface propensity are used to describe the RNA-binding specificity of the target residue. To accurately predict RNA-binding residues, our method adopts ensemble learning technique whose effectiveness in solving classification problems has been shown. The experimental results reveal that our method achieves a high performance on a benchmark test.

The clustering on 3-aa and 2-aa template patches show distinct structural patterns of RNA-binding sites. The clustering results indicate that RNAs have structure preference when binding with proteins. Currently these template patches are confined to 3-aa and 2-aa patches. In the future, we will extract larger template patches from binding sites and explore their structural patterns.

## Methods

### Dataset

The dataset used in our experiments is RB344 of PRIDB dataset [40]. RB344 is a non-redundant dataset which contains 344 proteins belonging to 13 categories: RNAse, SRP, aptamer, dsRNA, exosome, mRNA, ribosomal, small, snRNP, splicing, tRNA, viral, and other. Global sequence alignment was performed on the dataset using the needle function provided by the emboss suite [41]. The sequence identities in RB344 are smaller than 30 %. RNA-binding residues were determined using two definitions: (i) a residue whose any atom is within a 5 Å distance of any atom in a nucleotide; and (ii) residues involved in van der Waals, hydrogen-bonding, hydrophobic or electrostatic interactions with nucleotides [40]. Any amino acid residue satisfying the above definitions are regarded as RNA-binding residue. RNA-protein complexes in the dataset are shown in Table 4.

**Table 4:** Protein-RNA complexes in RB344 dataset

RNA category	PDB ID
RNAse	2BX2 2IX1 2NUG 2QKB 2XDB 2Y8Y 3BSU 3IAB 3T3O 3ULD 4 AM3 4ATO
SRP	1E8O 1HQ1 1JID 1LNG 1MFQ 2V3C 3KTW
Aptamer	1OOA 3AGV 3DD2 3V7E
dsRNA	1DI2 2YKG 3CIY 3EQT 3LRR 4IG8
Exosome	2JEA 2PO1 2VNU 4IFD
mRNA	1FXL 1GTF 1MSW 1UVM 1WPU 1WSU 1ZH5 2A8V 2F8K 2IPY 2J0S 2O5I 2PJP 2Q66 2VPL 2XGJ 2XNR 2XS2 2XZO 3BX2 3D2S 3I5X 3ICE 3MDI 3NMR 3P6Y 3PEY 3PO3 3Q0Q 3QGC 3R2C 3RER 4 F02 4HXH 4J7L 4JVY
Ribosomal	1DFU 1FEU 1FKA 1HR0 1I6U 1JBS 1MJI 1MMS 1MZP 1NKW 1SDS 1T0K 1UN6 1VQ8 1VQO 1Y69 2ASB 2BH2 2D3O 2 J01 2QA4 2VQE 2XFZ 2ZJQ 2ZJR 3AEV 3DH3 3F1E 3HUW 3I8I 3IEV 3KIS 3MOJ 3OIN 3R8S 3R8T 3R9X 3SFS 3SGF 3UMY 3 V24 3 V26 3V2C 3V2D 3V2F 3ZN9 4DH9 4GD1 4JUW 4JUX
Small	1SI3 1YVP 2BGG 2F8S 3A6P 3ADI 3HO1 3HTX 3 MJ0 3NMU 3NVI 3O7V 3VYX 3VYY 3ZC0 4F1N 4KRE
snRNP	1M8V 1URN 2OZB
Splicing	1A9N 2G4B
tRNA	1ASY 1B23 1C0A 1F7U 1FFY 1GAX 1H3E 1H4S 1J1U 1J2B 1K8W 1 N78 1Q2R 1QF6 1QTQ 1R3E 1SER 1U0B 1VFG 1WZ2 2AZX 2B3J 2CT8 2CZJ 2D6F 2DER 2DLC 2DU3 2FK6 2FMT 2GJW 2I82 2IY5 2ZNI 2ZUE 2ZZM 3AL0 3 AM1 3AMT 3BT7 3EPH 3FOZ 3HL2 3ICQ 3KFU 3OVB 3QSY 3TUP 3VJR 3W3S 3ZGZ 4ARC
Viral	1A34 1AV6 1DDL 1F8V 1HYS 1KNZ 1 N35 1PGL 1R9F 2AZ2 2BU1 2GIC 2GTT 2JLV 2QUX 2R7W 2W2H 2WJ8 2Z2Q 2ZI0 2ZKO 3AVX 3BSO 3KMQ 3 L25 3O8C 3RW6 3T5N 4FY7 4GV9 4H5P 4HKQ 4J1G 4K4Z
Other	1EC6 2ANR 2DB3 2GJE 2GXB 2PY9 2R8S 2XLK 3AF6 3HAX 3IEM 3PF4 3PKM 3QJL 3RC8 3S14 4B3G 4ERD 4FXD 4GG4 4ILL

PDB ID of protein-RNA complexes in RB344 dataset

### Identification of protein surface residues

To determine protein surface residues, accessible areas will be computed first. If its accessible area is larger than zero, the residue is considered as a surface residue. Otherwise it is a non-surface residue. The accessible areas can be calculated using VMD software [42] with the probe radius of 1.4 Å.

### Shape descriptor for protein residues

The backbone of an amino acid is defined by four atoms: N, CA, C, and O. The center of the side-chain is defined as the mean of coordinates of all heavy atoms on the side-chain. However, the side-chain of glycine only has a hydrogen substituent. Therefore, the hydrogen is used as the side-chain center of glycine. An amino acid is represented by atoms from its backbone and the point of its side-chain center.

### Template patches construction

The neighbors of a protein residue are defined as amino acids which are located within a certain distance (*d*_*N*_) on the protein surface. The distance between two amino acids is the smallest distance between their atoms. A patch is called 2-aa/3-aa patch if it is composed by two/three residues, respectively. Suppose a residue has a set of neighbors {*n*_1_, *n*_2_, …, *n*_*k*_}. When *k* ≥ 2, two neighbors can be selected from the neighbor set and construct a 3-aa patch with the residue. The total number of 3-aa patches constructed from the neighbor set is *C*2 *k*. When *k* = 1, a 2-aa patch can be constructed which is composed by the residue and its neighbor. The situation of *k* = 0 is not considered currently because we assume that interaction interfaces are areas consisting of two or more residues. If a surface residue in the training set is known to interact with RNAs, the patches constructed from its neighbors are regarded as positive patches. Only positive patches are used as template patches. We obtained 175,989 3-aa template patches and 122 2-aa template patches from RB344 dataset when *d*_*N*_ equals 3 Å.

### Structural similarity between patches

Because each amino acid can be represented as a set of atoms from the backbone and the center of its side chain, a 3-aa/2-aa patch can be represented as the assembly of representative points from all its member residues. When comparing shapes of two surface patches of the same size, i.e. both of them are 3-aa or 2-aa patches, they are treated as rigid objects. The structural similarity between two patches can be measured by the sum of Euclidean distances between the corresponding points after rotation and translation (i.e. the least-squares distance between two sets of points). Suppose a patch contains *m* (*m*∈{2,3}) residues, each of which is composed by *n* points, the least-squares distance between patch *X* and *Y* can be computed using Eq. (1).

1 $$ {d}_{LS}\left(X,Y\right)=\sqrt{\underset{s,R,t}{ \min }{\displaystyle \sum_{i=1}^m{\displaystyle \sum_{j=1}^n}{\left\Vert {x}_{ij}-\left(sR\left({y}_{ij}\right)+t\right)\right\Vert}^2}} $$

where *s* is a scale factor, *R* is a rotation matrix, and *t* is a translation vector. *x*_*ij*_ and *y*_*ij*_ are the *j*-th point from *i*-th residue of *X* and *Y* respectively. The optimal solution of *s*, *R*, and *t* for Eq. (1) is:

2 $$ R=V{U}^T,\;s=\sqrt{\frac{{\displaystyle \sum_{i=1}^n}{\left\Vert x{\hbox{'}}_i\right\Vert}^2}{{\displaystyle \sum_{i=1}^n}{\left\Vert y{\hbox{'}}_i\right\Vert}^2}},\;t=\overline{x}-sR\left(\overline{y}\right) $$

In Eq. (2), $\overline{x}$ and $\overline{y}$ are the centroids of *X* and *Y*. Matrices *U* and *V* are obtained by singular value decomposition: *Y*'*X*'^*T*^ = *UΣV* ^*T*^, where *X*' = {*x*'_*ij*_}_*i*=1,…,*m*; *j*=1,…,*n*_ and *Y*' = {*y*'_*ij*_}_*i*=1,…,*m*; *j*=1,…,*n*_ are obtained by subtracting $\overline{x}$ and $\overline{y}$ from the points, i.e. $x{\hbox{'}}_{ij}={x}_{ij}-\overline{x}$ and $y{\hbox{'}}_{ij}={y}_{ij}-\overline{y}$; *i* = 1,…,*m* and *j* = 1,…,*n*. Details of this optimal solution can be found in [43]. In our problem, we assume that there is no scale change between two similar patches. Therefore, the scale factor *s* is set to 1 instead of using the value in Eq. (2).

To compute the least-squares distance, the correspondence between points from two objects should be known in advance. However, the correspondence between the residues from two patches has not been determined yet. Therefore, orders of residues in patch *X* are permuted to create different correspondences to residues in patch *Y*. Once the correspondence between residues from two patches has been determined, the correspondence between their representative points will be automatically determined. To compare two 3-aa patches, there are 6 ways (*P*3 3) of correspondences and to compare two 2-aa patches, there are 2 ways (*P*2 2) of correspondences. We compute the least-squares distances between two patches using different correspondences and the minimum one is defined as the structural similarity (*d*_*SS*_) between two patches. *d*_*SS*_ can be computed using Eq. (3) according to its definition.

3 $$ {d}_{SS}\left(X,Y\right)=\underset{i\in \left\{1,2,\cdots, {P}_m^m\right\}}{ \min }{d}_{LS}\left({X}^{(i)},Y\right) $$

In Eq. (3), *X* ^(*i*)^ is the *i*-th way of reordering residues in patch *X* and there are *Pm m* ways (*m* is the number of residues in a patch) of residue reordering in all.

### Clustering template patches

**Fig. 3:**
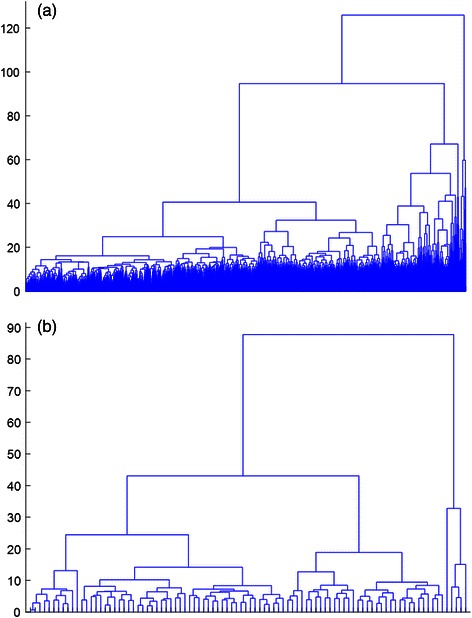
Hierarchical clustering on 3-aa and 2-aa template patches. Hierarchical clustering with complete-linkage on (**a**) 3-aa and (**b**) 2-aa template patches

It’s difficult to use all template patches to construct structural features. Therefore, we select some representative ones from them so that the dimension of structural features can be acceptable. We group the extracted 3-aa and 2-aa template patches using complete-linkage hierarchical clustering. The distance metric used in clustering algorithm is the least-squares distance shown in Eq. (3). The cluster dendrograms of 3-aa patches and 2-aa patches extracted from the training set in a fold of cross validation are shown in Fig. 3. Hierarchical clustering using single- and average-linkage is also performed. However, the resulting dendrograms have ladder shapes. It indicates that these two methods are not suitable for clustering template patches. The final clusters represent distinct structural patterns of template patches. They are more or less similar to protein structural motifs but are much smaller. They can be regarded as binding units of interaction interfaces of proteins and are used to describe RNA-binding surfaces.

In each fold of cross validation, there are ~130,000 3-aa template patches constructed from the training set. We randomly selected 10,000 3-aa template patches and performed hierarchical clustering. The selected 3-aa patches and all 2-aa patches are further grouped into 40 and 20 clusters. In each cluster, the centroid patch, which has the smallest sum-of-square distance to other members, is also determined. The centroid patches are regarded as the representative patches.

**Fig. 4:**
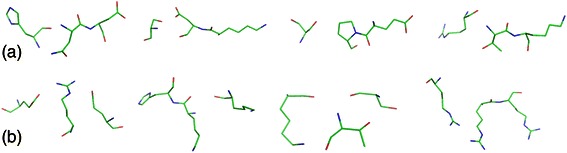
Structures from two clusters of 3-aa template patches. **(a)** Four template patches in Cluster 1; **(b)** four template patches in Cluster 4

Patches in each cluster reveal distinct structural patterns. For example, in one cluster, three amino acids are arranged in a linear way (see Fig. 4(a)). While in another, they are placed like the head of a fork (see Fig. 4(b)). The sequences of patches in each cluster are not conserved. However, their structures are quite similar. It indicates that template patches have specific structural patterns and RNAs may have structure preference when binding with proteins.

### The new structural features construction

Given a residue *r*, structural features can be constructed in the following way. Firstly, all neighbours of *r* located within the distance of *d*_*N*_ on the protein surface are identified. Suppose there are *k* neighbouring residues and they are denoted by {*n*_1_, *n*_2_,…, *n*_*k*_}. A set of patches {*X*_1_,*X*_2_,…,*X*_*K*_} can be constructed using {*n*_1_, *n*_2_,…, *n*_*k*_} and *r*: if *k* = 1, i.e. *r* has only one neighbour *n*_1_, a 2-aa patch {*X*_1_} which is simply composed of *r* and *n*_1_ can be constructed. If *k* ≥ 2, several 3-aa patches which are composed of *r* and two of its neighbours can be constructed (the total number of 3-aa patches, *K*, equals to ${C}_k^2$). To construct structural features for residue *r*, {*X*_1_,*X*_2_,…,*X*_*K*_} will be compared with each representative patch and accumulated distances to each representative patch will be computed.

Denote the set of representative patches by {*Y*_1_,*Y*_2_,…,*Y*_*L*_}. The length of the new feature vector *L* is the total number of 3-aa and 2-aa representative patches. In our work, *L* is 60 (because there are 40 3-aa representative patches and 20 2-aa representative patches). Rearrange representative patches and let {*Y*_1_,*Y*_2_,…,*Y*_40_} be 3-aa representative patches and {*Y*_41_,*Y*_42_,…,*Y*_60_} be 2-aa representative patches.

If *k* = 1, there will be only one 2-aa patch {*X*_1_} surrounding *r. X*_1_ will be compared with each 2-aa representative patch. Suppose *f*_*j*_ is the distance of *X*_1_ to *Y*_*j*_ (40 < *j* ≤ *L*). Then

4 $$ {f}_j=\left\{\begin{array}{cc}\hfill {d}_{SS}\left({X}_1,{Y}_j\right)\hfill & \hfill 40<j\le L\hfill \\ {}\hfill 0\hfill & \hfill 1\le j\le 40\hfill \end{array}\right. $$

Because *X*_1_ only contains two residues, it cannot be compared with 3-aa representative patches. The distances between *X*_1_ and 3-aa representative patches are directly set zeros.

If *k* ≥ 2, each 3-aa patch *X*_*i*_ (*i* = 1,…,*K*) is compared with *Y*_*j*_ (1 ≤ *j* ≤ 40). *f*_*j*_ can be computed using Eq. (5).

5 $$ {f}_j=\left\{\begin{array}{cc}\hfill {\displaystyle \sum_{i=1}^K{d}_{SS}\left({X}_i,{Y}_j\right)}\hfill & \hfill 1\le j\le 40\hfill \\ {}\hfill 0\hfill & \hfill 40<j\le L\hfill \end{array}\right. $$

*f*_*j*_ (*j* = 1,…,40) is the accumulated distance of surrounding patches {*X*_1_, *X*_2_,…, *X*_*K*_} to the representative patch *Y*_*j*_. When 40 < *j* ≤ 60, *f*_*j*_ is set zeroes because *X*_*i*_ is a 3-aa patch which cannot be compared with 2-aa representative patches. In the end, a 60-dimension feature vector [*f*_1_,…,*f*_*L*_] can be constructed for the residue *r*.

The rationale of comparing {*X*_1_,*X*_2_,…, *X*_*K*_} with representative patches is as follows. The protein surface around a binding residue can be characterized by template patches. After clustering, template patches can be approximated by representative patches. Therefore, we can describe the protein surface surrounding a binding residue using the combination of representative patches. The problem is how to quantitatively measure the structural similarity of surfaces surrounding two residues. Considering that surfaces can be approximated by combinations of representative patches, we compute the accumulated distance of surrounding patches to each representative patch and denote it as a structural feature. If there are *L* representative patches, *L* features will be obtained. These structural features contain potential structural information. It can be seen that, for all residues, no matter RNA-binding or non-RNA-binding, their structural features can be constructed by computing the accumulated distance of surrounding patches to representative patches. Given a target residue *r*, if its surrounding surface is similar to the surfaces surrounding RNA-binding residues, its structural features will be more close to features of RNA-binding residues. Based on its structural features, *r* can be classified as an RNA-binding residue or a non-RNA-binding residue.

### Other features used for RNA-binding residue prediction

In addition to the proposed structural features, other sequence features of amino acids are also introduced to describe RNA-binding property. Each residue in RNA-binding proteins is characterized by another two descriptors including: (i) PSSM which gives values of sequence conservation for residues using PSI-BLAST [44]; (ii) the residue interface propensity which describes the frequency of different types of amino acids occurring in the interaction interface than on the protein surface [23].

For residues from the training set and the test set, we can construct feature vectors which combine the new structural features and two additional sequence features. The dimension of all features is 81.

### RNA-binding residue prediction using ensemble method

RNA-binding residue prediction can be regarded as a classification problem when feature vectors have been presented. In the learning process, a classification model can be learned using feature vectors and class labels of residues from the training set. Then, the classification model can be applied to predict binding propensities for residues in the test set. Compared with individual classifiers, ensemble classifiers have already been shown to produce better classification results [45, 46]. Specifically, in the problem of RNA-binding residue prediction, random forest, an ensemble classifier, has already been adopted and showed a high performance.

In our method, ensemble learning technique is also used. ENTOOL [47] is a package which integrates a series of classification algorithms, which include SVM, decision tree, ridge regression, Gaussian mixture models, multilayer perceptron, etc. In our work, models of ridge regression, perceptron, and multilayer perceptron are selected as constituent classifiers because they can achieve better performances than other classifiers in ENTOOL.

### Methods for prediction performance evaluation

ENTOOL first performs five-fold cross-validation on the training residues to adjust parameters of the ensemble classifier and then predicts binding scores for target residues using the trained models. The predicted binding scores vary from −1 to 1. The larger the binding score, the higher binding propensity of the target residue.

By comparing the predicted scores with the true labels of those residues in the test set, four metrics can be computed: true positives (TP), true negatives (TN), false positives (FP), and false negatives (FN). Based on the four metrics, false positive rate (FPR) and true positive rate (TPR, which is also called sensitivity) can be computed (see Eq. (6)). ROC curve can be created by plotting FPR values against TPR values. Other performance metrics, such as AUC, accuracy, precision, specificity, F-score, and MCC can also be computed (see Eq. (6)).

6 $$ \begin{array}{l}\mathrm{T}\mathrm{P}\mathrm{R}=\mathrm{sensitivity}=\frac{\mathrm{TP}}{\mathrm{TP}+\mathrm{F}\mathrm{N}}\;\\ {}\mathrm{F}\mathrm{P}\mathrm{R}=\frac{\mathrm{FP}}{\mathrm{TN}+\mathrm{F}\mathrm{P}}\\ {}\mathrm{accuracy}=\frac{\mathrm{TP}+\mathrm{T}\mathrm{N}}{\mathrm{TP}+\mathrm{F}\mathrm{P}+\mathrm{F}\mathrm{N}+\mathrm{T}\mathrm{N}}\\ {}\mathrm{precision}=\frac{\mathrm{TP}}{\mathrm{TP}+\mathrm{F}\mathrm{P}}\\ {}\mathrm{specificity}=\frac{\mathrm{TN}}{\mathrm{TN}+\mathrm{F}\mathrm{P}}\\ {}\mathrm{F}\hbox{-} \mathrm{score}=\frac{2\mathrm{T}\mathrm{P}}{2\mathrm{T}\mathrm{P}+\mathrm{F}\mathrm{P}+\mathrm{F}\mathrm{N}}\\ {}\mathrm{M}\mathrm{C}\mathrm{C}=\frac{\mathrm{TP}\times \mathrm{T}\mathrm{N}-\mathrm{F}\mathrm{P}\times \mathrm{F}\mathrm{N}}{\sqrt{\left(\mathrm{T}\mathrm{P}+\mathrm{F}\mathrm{N}\right)\left(\mathrm{T}\mathrm{P}+\mathrm{F}\mathrm{P}\right)\left(\mathrm{T}\mathrm{N}+\mathrm{F}\mathrm{P}\right)\left(\mathrm{T}\mathrm{N}+\mathrm{F}\mathrm{N}\right)}}\end{array} $$

## Abbreviations

AUC: Area under the curve
FN: False negatives
FP: False positives
FPR: False positive rate
MCC: Matthews correlation coefficient
PSSM: Position-specific scoring matrix
ROC: Receiver operating characteristic
SVM: Support vector machine
TN: True negatives
TP: True positives
TPR: True positive rate
